# Triple Nerve Analgesia Block for Facial Dog-bite Laceration in a Child

**DOI:** 10.4274/TJAR.2025.241827

**Published:** 2026-02-09

**Authors:** Bheemas B. Atlapure, Mahammad Azeez Aspari, Dalim Kumar Baidya, Habib M. Reazaul Karim

**Affiliations:** 1 All India Institute of Medical Sciences Guwahati, Department of Anaesthesiology Critical Care and Pain Medicine, Guwahati-Assam, India

**Keywords:** Child, face surgery, nerve block, postoperative analgesia

Dear Editor,

Multiple superficial nerves supply the face, and peripheral nerve blocks play an essential role in multimodal analgesia. Infraorbital nerve blocks are valuable for upper lip and nose surgeries, such as cleft lip repair, septoplasty, and nasal endoscopy.^1, 2^ The mental nerve provides sensory supply to the lower lip, skin, and buccal mucosa ventral to the mental foramen. Maxillary nerve blocks allow for anaesthesia over cosmetically significant areas of the cheek without causing local wound oedema, facilitating repair.^3^ However, multiple nerve blocks in paediatric facial surgeries, especially in a single patient and given the highly vascularised nature of face, are rarely practised and reported. Nevertheless, combining these three blocks may be necessary in unique cases. We performed a triple nerve block on a 2-years-old, 12 kg boy with facial lacerations caused by a dog-bite affecting the left mouth angle, philtrum, infraorbital, and maxillary areas (Figure 1).

Following parental counselling and informed consent, the case was operated under general anaesthesia, and each block was performed using the following landmark-based techniques: i) Infraorbital nerve block: A 25-gauge needle was advanced at the infraorbital foramen, limiting advancement by palpating the foramen, and 2 mL of local anaesthetic (LA) was injected after negative aspiration. ii) Mental nerve block: A 25-gauge needle was inserted near the mental foramen, directed laterally to medially, and 2 mL of LA was injected after confirming no blood return. iii) Supra-zygomatic maxillary nerve block: The needle entry was located at the angle of the zygomatic arch and posterior orbital rim. A 25-gauge needle was inserted perpendicularly to reach the greater wing of the sphenoid at a depth of 10 mm, then reoriented caudally and posteriorly, advancing 20 mm to reach the pterygopalatine fossa, where 2 mL of LA was injected after negative aspiration. A total of 6 mL of LA was used, comprising 3 mL of 0.25% bupivacaine, 3 mL of 1% lidocaine with 1:200,000 epinephrine, and 1 mg of dexamethasone. Intraoperatively, intravenous paracetamol was administered at a dose of 15 mg kg^-1^ as part of a multimodal analgesia regimen. Postoperatively, paracetamol was continued at 15 mg kg^-1^ every 8 hours for 2 days. Postoperatively, the child remained calm, cooperative, and non-agitated in the post anaesthesia care unit, pain-free for 48 hours without requiring any further rescue analgesia and resumed oral intake within a day.

A recent study suggests that these blocks may be especially beneficial in paediatric patients, offering pain relief without systemic opioid-related side effects.^4^ Nonetheless, meta-analysis also indicates that the benefits of these blocks go beyond pain control and provide benefits against agitation.^2^

The practice of multiple nerve blocks for facial trauma and lacerations is rare, and the reported median (interquartile range; Q3-Q1 of a number of regional nerve blocks is shown to be 1 (1-1).^5^ The present case hints that the triple nerve block approach may be considered for perioperative pain management in cases with multiple facial lacerations. We will require more cases in the future to ascertain the safety and efficiency.

## Figures and Tables

**Figure 1:**
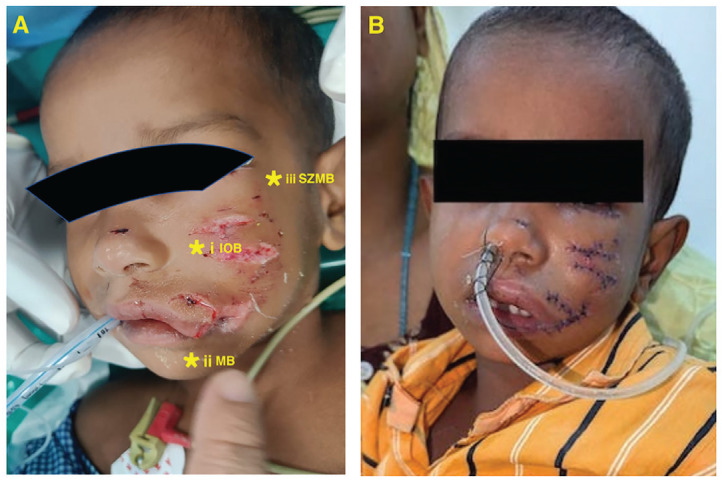
Dog-bite lacerations of the left cheek and lip in a 2-year-old boy. A (Pre-repair): Wounds before surgical debridement and closure, with planned regional anaesthetic sites marked by yellow asterisks—i: infra-orbital nerve block, ii: mental nerve block, iii: supra-zygomatic maxillary block—. B (Post-repair): Immediate postoperative appearance showing layered suturing and a nasotracheal tube *in situ*. IOB, infra-orbital nerve block; MB, mental nerve block; SZMB, supra-zygomatic maxillary block.
